# Stress exposure in early post-natal life reduces telomere length: an experimental demonstration in a long-lived seabird

**DOI:** 10.1098/rspb.2013.3151

**Published:** 2014-05-07

**Authors:** Katherine A. Herborn, Britt J. Heidinger, Winnie Boner, Jose C. Noguera, Aileen Adam, Francis Daunt, Pat Monaghan

**Affiliations:** 1 Institute of Biodiversity, Animal Health and Comparative Medicine, College of Medical, Veterinary and Life Sciences, University of Glasgow, Graham Kerr Building, Glasgow G12 8QQ, UK; 2 Centre for Ecology & Hydrology, Bush Estate, Penicuik, Midlothian EH26 0QB, UK

**Keywords:** ageing, stress, telomere, senescence, lifespan, *Phalacrocorax aristotelis*

## Abstract

Exposure to stressors early in life is associated with faster ageing and reduced longevity. One important mechanism that could underlie these late life effects is increased telomere loss. Telomere length in early post-natal life is an important predictor of subsequent lifespan, but the factors underpinning its variability are poorly understood. Recent human studies have linked stress exposure to increased telomere loss. These studies have of necessity been non-experimental and are consequently subjected to several confounding factors; also, being based on leucocyte populations, where cell composition is variable and some telomere restoration can occur, the extent to which these effects extend beyond the immune system has been questioned. In this study, we experimentally manipulated stress exposure early in post-natal life in nestling European shags (*Phalacrocorax aristotelis*) in the wild and examined the effect on telomere length in erythrocytes. Our results show that greater stress exposure during early post-natal life increases telomere loss at this life-history stage, and that such an effect is not confined to immune cells. The delayed effects of increased telomere attrition in early life could therefore give rise to a ‘time bomb’ that reduces longevity in the absence of any obvious phenotypic consequences early in life.

## 1. Introduction

Exposure to various stressors during early life can have profound fitness consequences [1–3]. In some cases, such early life adversity can give rise to a phenotype whose performance is impaired throughout life [4]. However, in other cases, the phenotypic consequences do not become evident until much later in life, sometimes decades later, when the individuals begin to show signs of accelerated ageing [5]. Although understanding the relationship between early life conditions and longevity has important implications for diverse biological fields, we still have very little information about the mechanisms involved in producing links over such long time scales. A key process that could produce long lasting, but delayed, effects is stress-induced variation in telomere attrition [6–8]. Telomeres are highly conserved, tandem repeats of a short DNA sequence (generally TTAGGG in vertebrates), which, together with protein complexes, form a protective cap at the ends of eukaryote chromosomes. Telomeres have a number of functional roles in genome stability and replication. Particularly important among these are that telomeres protect the coding sequences of the genome from the loss that occurs owing to incomplete replication of the lagging strand during DNA replication, and prevent chromosome ends from being misidentified as double-stranded breaks by the DNA repair machinery. When telomeres reach a critically short length, cells enter a state of replicative senescence following which they either die, or show an altered secretory profile and produce inflammatory compounds. Both effects contribute to the decline in tissue function that occurs in later life [9]. The magnitude of telomere loss with each round of cell division is generally greater than that attributable to the DNA replication process and is influenced by conditions within the cell. Telomeres are particularly sensitive to damage induced by oxidative stress, which has been shown to be associated with increased telomere loss during cell replication both *in vivo* and *in vitro* [10–13].

During post-natal life, the average length of telomeres in host somatic cell populations declines with age in many vertebrates, and both starting telomere length and the rate of loss differ among individuals. Several studies in humans and other species have shown that telomere length at a given age is correlated with future life expectancy [14]. In a detailed, longitudinal study of telomere length and longevity covering the entire lifespan of a group of zebra finches, we showed that telomere length early in life is the best predictor of eventual lifespan; the predictive power of telomere length measured later in life was weaker, and largely a consequence of its strong correlation with early life telomere length [15]. Therefore, a particular important question is, does early life adversity influence early life telomere length?

A number of recent studies have shown that exposure to various stressors is associated with reduced telomere length [6,16]. Mostly, these studies involve humans and the telomere measurements have been made in leucocytes. The results of these studies generally support a link between elevated stress hormones, oxidative stress and reduced telomere length [7]. However, white blood cells comprise a complex of immune cell types with different turnover rates that show distinct rates of telomere erosion [17]. A number of mammalian white cell types also express the enzyme telomerase, which can restore telomere sequences; this enables rapid local cell proliferation in response to acute infection [17]. Changes in the proportion of cell types could therefore influence estimates of telomere length. Both correlative and experimental studies have shown that there is some upregulation of telomerase in white blood cells in response to environmentally induced stress, which may mitigate the effect on telomere loss [18,19]. It is possible that the reported effects of stress on telomere length might be confined to cells of the immune system because of their particular proliferative profiles [9]. It is therefore important that we know whether stress exposure affects telomere length in cells outside the immune system.

Human studies of stress and telomere dynamics are generally correlative, making it difficult to separate cause and effect for several reasons. Because conditions early and later in life are likely to be correlated, it is not possible to distinguish between effects that are due to the current environment from those due to developmental conditions. Particular phenotypes that have shorter telomere lengths might also be more likely to encounter environmental stressors, or respond more strongly to them. In human studies, the telomere measurements have generally been made in adults and mostly related to current stressor exposure [9]. Where a link has been made to conditions during early life, these links have generally been assessed retrospectively, with individuals self-reporting the level of childhood stress experienced, which may be unreliable. Without an early life telomere measurement, we cannot ascertain whether early adversity affected telomere length at that time, or resulted in individuals more susceptible to factors that accelerate telomere loss in later life as a consequence of their early life conditions [2,9]. A recent correlative human study has linked pre-natal exposure to stressors to reduced leucocyte telomere length at birth [20]. This effect is supported by an experimental study in chickens, in which stress hormone levels were increased in eggs; the resulting chicks showed reduced telomere length measured in erythrocytes 25 days after hatching [21]. In pre-natal studies, the stress hormones to which the embryo is initially exposed are, at least initially, of maternal origin, and the activity of the enzyme telomerase, which can restore telomere length, is generally much higher during embryonic development than in post-natal life [22,23]. Thus, the effect of elevated stress during the embryonic period may be different from its post-natal effect. As yet however we do not know whether post-natal stress affects telomere length.

Our aim in this study was to examine experimentally the effect of stress exposure on telomere loss during early post-natal life. We used wild birds in which it is possible to measure within-individual changes in telomere length in red blood cells over time. Since avian red blood cells are nucleated and do not express telomerase [24], use of these cells avoids the possible complications associated with interpreting results from cells of the immune system. By working with a free-living population, individuals in our treatment groups encompassed the natural range of variation in their genetic make-up, their stress responsiveness and the environmental conditions that individuals experience during growth, all of which can be altered in captive populations. Thus, we were able to assess the extent to which the increased exposure to stress that we imposed can have a significant effect on telomere loss over and above these other sources of variation. We used two ways of applying repeated exposure to a stressor. The first was one in which birds were handled daily for 20 days during their main growth period—which elicits anti-predator responses in the parents and elevates stress hormone levels in the chicks. The second was one in which the handling of the chicks was combined with an experimental elevation of corticosterone (CORT, the main glucocorticoid stress hormone in birds), which elevated stress hormone levels within the natural range. We also had a control group where no handling or hormone administration took place. We checked that our experiment was biologically meaningful by establishing that the chicks are naturally capable of mounting a hormonal response to external stressors, and that the CORT increase induced by our hormone manipulation was within the naturally occurring range in both treatment groups. We also examined the effect of the manipulation on the stress response itself, as increased stress reactivity is the route whereby an effect on telomere attrition is most likely. Our results show unequivocally that increased exposure to a stressor during early post-natal life results in a reduction in telomere length at this life-history stage.

## 2. Material and methods

### (a) Study subjects

This study was conducted between May and July 2012, on a free-living population of a socially monogamous, relatively long-lived (14+ years) cormorant species, the European shag (*Phalacrocorax aristotelis*). The study population breeds on the Isle of May National Nature Reserve (56° 11′ 9″ N, 2° 33′ 27″ W), a 110 acre island, approximately 5 miles off the coast of Scotland in the Firth of Forth. Females lay clutches of one to five eggs; the modal clutch size in this population is three eggs. The altricial chicks hatch asynchronously, with hatching intervals varying among nests. Chicks remain in the nest until fully grown, aged approximately 55 days. During this time, they can be subjected to a number of environmental stressors that have been shown to affect growth rate or survival, including predator presence, sibling competition, inclement weather and reduced food availability.

### (b) Natural stress responsiveness of subjects

The age at which developing individuals are capable of mounting a hormonal stress response varies among species [25]. Therefore, to establish that 10-day-old shag chicks were able to respond to stressors by elevating CORT levels, we exposed a separate sample of chicks (*n* = 16 chicks from six nests), not used in our main experiment, to a standardized capture and restraint protocol (held in an opaque, cloth bag [26]). Chicks were captured at the nest and a small blood sample collected from the tarsal vein within 3 min of capture to give a baseline measure. The actual time required to collect the sample (hereafter termed ‘bleed time’) was recorded. Chicks were then placed in opaque, cloth bags and subsequent blood samples collected after 10 and 20 min to record changes in CORT. CORT levels significantly increased in response to handling time (*F*_2,28.47_ = 20.47, *p* < 0.001; mean ± 1 s.e.m.: baseline = 18.85 ± 2.58 ng ml^−1^, 10 min = 40.15 ± 4.94 ng ml^−1^, 20 min = 52.57 ± 6.25 ng ml^−1^), confirming that 10-day-old shag chicks can mount a stress response and are consequently naturally exposed to variation in CORT levels.

### (c) Experimental treatment

Nests (*n* = 51) were located during laying and monitored from a distance every 4 days to minimize disturbance. When one or more chicks were first seen in a nest, hatch date was assigned directly if hatching was observed, otherwise estimated from wing length [27]. The nest was then left undisturbed until the first hatched chick was approximately 10 days old (age range = 5–13 days, mean = 9.39, s.e. = 0.13, hereafter day 10 of the experiment). To measure growth and telomere length prior to administration of experimental treatments, all of the chicks were captured and bled, and the nest was randomly assigned to one of the following treatment groups: unhandled (*n* = 36 chicks from 17 nests), handled-oil (*n* = 36 chicks from 16 nests) or handled-CORT (*n* = 42 chicks from 18 nests), with all chicks within a nest receiving the same treatment. In a subset of chicks in each treatment (unhandled *n* = 15 chicks from 13 nests, handled-oil *n* = 10 chicks from nine nests, handled-CORT *n* = 12 chicks from 12 nests), the first blood sample was collected within 3 min (with the bleed time recorded) to measure baseline CORT levels. From day 10 until day 29, chicks in the handled-CORT treatment received daily oral doses of 0.5 μg g^−1^ of CORT (C2505; Sigma-Aldrich, Dorset, UK) dissolved in cod liver oil (Carlson Laboratories, Arlington Heights, IL, USA) at a concentration of 2 mg ml^−1^. The CORT mixture was sonicated daily to ensure that CORT remained evenly in solution. Oral doses were administered using a pipette. To minimize disturbance at the nest, the volume was adjusted throughout the experiment for average mass gain based on previously collected data on natural variation in chick growth from 262 chicks measured in 2010 and 2011, ranging from 19 μl at day 10 to 645 μl at day 29. This methodology allowed us to avoid measuring each chick daily and to prepare the doses in advance. The CORT dose was based on other studies that have orally manipulated CORT levels in developing altricial chicks [28]. Chicks in handled-oil nests received equivalent daily oral doses of only the fish oil during this time. The nests in the unhandled group were not visited between days 11 and 29. In the two experimental groups, chicks within the broods were individually identified by tagging. In the control group, which were not handled, chicks were identified based on the stable size differences that occur in chicks in shag broods as a consequence of variation in egg size and hatching asynchrony; the stability of these size differences until 30 days of age was verified in the tagged broods.

To verify that the administration of CORT in the handled-CORT chicks did indeed elevate CORT levels, a second blood sample was collected from a sub-sample of chicks from the handled-oil and handled-CORT groups either 10 min (handled-oil *n* = 13 chicks from 11 nests, handled-CORT *n* = 17 chicks from 16 nests) or 30 min (handled-oil *n* = 10 chicks from nine nests, handled-CORT *n* = 10 chicks from nine nests) after the first administration of the oral treatments on day 10. On day 30 (30 days after the experiment began when the first hatched chick was approximately 30 days old, age range = 26–33 days, mean = 29.65, s.e.m. = 0.14), all of the chicks within each nest were blood sampled to examine the effect of the treatment on CORT levels and telomere length. An initial blood sample was collected within 3 min (with bleed time recorded), to examine baseline CORT levels and telomere length. Chicks were then subjected to the standardized capture and restraint protocol (described above) and a subsequent blood sample was collected after 10 min, to measure stress-induced CORT levels. Chicks were then weighed, measured and returned to the nest. All blood samples were stored on ice for less than 6 h, centrifuged, and plasma and erythrocytes were separated and stored at –80°C until hormone and telomere analyses.

Our CORT treatment successfully elevated hormone levels within the natural range; the mean CORT level of handled-CORT chicks was within 2 s.d. of the mean CORT levels in the group of unmanipulated chicks exposed to standardized capture and handling stress at 10 days (see §2*b*). Furthermore, the highest CORT level of handled-CORT chicks (165 ng ml^−1^) was lower than the maximum level induced by capture and handling stress in 10-day-old chicks in this population (235 ng ml^−1^). The handling was itself stressful, but the oral administration of oil did not add to the stress experienced by the handled-oil birds as measured by CORT levels. CORT levels in the handled-oil birds, though not as high as the handled-CORT birds, were not significantly different from levels observed in response to standardized capture and handling stress at 10 days (see above) (*F*_1,14.42_ = 0.037, *p* = 0.85, mean ± 1 s.e.m.: maximum CORT in response to capture and handling stress = 56.22 ± 7.11 ng ml^−1^).

### (d) Corticosterone measurement

CORT levels were measured using an enzyme-immunoassay (EIA) kit (Assay Designs Corticosterone Kit 901–097, Enzo Life Sciences, Exeter UK). Serially diluted shag chick plasma ran parallel to the standard curve of the EIA kit, indicating that the kit could be used to measure CORT in European shags. To measure extraction efficiencies, 15.75 μl plasma samples were equilibrated with 20 μl of 150 cpm ^3^H CORT. Samples were then extracted twice with 1 ml of diethyl ether, snap frozen in a dry ice and methanol bath, and the supernatant was transferred to a new tube. Samples were then dried down under nitrogen gas and resuspended in 300 μl of assay buffer and left in the refrigerator overnight. The following day, 50 μl of each sample was placed in a scintillation vial with 1 ml of Ecoscint scintillation fluid (National Diagnostics, Atlanta, GA, USA) and counted on a scintillation counter to measure extraction efficiencies. Samples were run in duplicate and controls (blanks, non-specific binding, maximum binding and total activity) were run in triplicate according to the manufacturer's instructions, with the following modification: our standard curve contained the following points: 20 000, 4000, 1000, 250, 62.5, 15.63 pg ml^−1^. Plates were read on a spectrophotometer at 405 nm and corrected to 570 nm. CORT concentrations were determined using the curve fitting program AssayZap (Biosoft, Cambridge, UK). Final CORT values were corrected for extraction efficiencies. The same, pooled, shag chick plasma sample was included on every plate to measure inter-assay variation. All samples collected from the same individual on the same day as part of a stress series were included on the same plate, and samples from the three experimental treatment groups were balanced across 17 plates. The average extraction efficiency was 85%, the average intra-assay variation was 8% (range 6–12%) and the inter-assay variation was 15%.

### (e) Telomere measurement

Telomere length was measured in red blood cells (RBC), a highly proliferative, nucleated tissue in birds, well suited to telomere analyses. When chicks were blood sampled more than once on the same day, telomere measurements were made on the first blood sample collected. DNA was extracted from 4 μl samples of RBC in 196 μl of phosphate-buffered saline using Macherey-Nagel Whole Blood Kits (Macherey-Nagel, Bethlehem, PA, USA) and following the manufacturer's instructions. We measured the quantity and purity of the extracted, genomic DNA using a Nanodrop 8000 spectrophotometer (Thermo Scientific) and measured telomere length using quantitative PCR (qPCR) on an Mx3005P (Stratagene), which provides a relative measure of telomere length suitable for within species comparisons [29,30]. Telomere length was measured as the ratio (T/S) of telomere repeat copy number (T) to control, single gene copy number (S), relative to a reference sample. The control, single copy gene used was *glyceraldehyde-3-phosphate dehydrogenase* (*GAPDH*). We followed the methods described by Cawthon [31], adapted for European shags as follows. Ten nanogram of DNA was used for both the telomere and GAPDH reactions. The following primers were used to amplify the telomere and *GAPDH* sequences: Telomere *forward tel1b* (5′-CGGTTTGTTTGGGTTTGGGTTTGGGTTTGGGTTTGGGTT-3′) and *reverse tel2b* (5′-GGCTTGCCTTACCCTTACCCTTACCCTTACCCTTACCCT-3′); Cormorant specific *GAPDH* *forward* (GACTGTAGCCTTCTCCTTCCCTTA) and *reverse* (TTCCCATCCACTTCCAGTAAAGA). We used the following primer concentrations: *forward tel1b/reverse tel2b* 200/200 nM and *forward GAPDH*/*reverse GAPDH* 200/200 nM. Primers were mixed with 12.5 μl of absolute blue SYBR green QPCR Master Mix (Stratagene) for a total volume of 25 μl. Telomere and GAPDH reactions were carried out on separate plates. The conditions for the qPCR reactions were: *telomeres* 15 min at 95°C, followed by 27 cycles of 15 s at 95°C, 30 s at 58°C and 30 s at 72°C; *GAPDH* 15 min at 95°C, followed by 40 cycles of 15 s at 95°C, 30 s at 60°C and 30 s at 72°C. In both reactions, the number of PCR cycles (*C*_t_) necessary to accumulate sufficient fluorescent signal to cross a threshold was measured and individuals with relatively long telomeres were characterized by shorter reaction times.

A shag chick RBC reference sample was serially diluted to produce a standard curve on every plate to measure the reaction efficiencies. All of the samples fell within the bounds of the standard curve and the mean reaction efficiencies were 100.8 (range 98.85–105) for GAPDH and 101.5 (range 97.2–105) for telomeres. This same reference sample was also used to set the *C*_t_ thresholds for the reactions and to calculate intra- and inter-plate variation. Samples and standard curves were run in triplicate, and the mean values were used to calculate the relative T/S ratios for each sample using the following formula: 2^ΔΔCt^, where ΔΔ*C*_t_ = (*C*_t_^telomere^ – *C*_t_^GAPDH^) reference – (*C*_t_^telomere^ – *C*_t_^GAPDH^) focal [32]. Mean intra- and inter-plate variation of the *C*_t_ values was 0.97 and 2.2% for the telomere reactions and 0.38 and 0.65% for the GAPDH reactions.

### (f) Statistical analyses

Linear mixed effects (LME) models were used for all analyses. The variance structures were estimated using restricted maximum likelihood (REML) and all models had normal error structures. Nest was included as a random factor in all models to account for the non-independence of chicks from the same nest. We examined the potential effects of the treatment on baseline CORT levels and telomere length at the first sampling point (i.e. when the first hatched chick in the nest was around 10 days old), and on baseline and handling stress-induced CORT levels (sample collected 10 min after capture and restraint) and telomere length at the second sampling point (i.e. at the end of the experiment when the first hatched chick in the nest was around 30 days old). In these models, we also included sex and hatching rank as fixed factors, and age, body mass and brood size as covariates. Models of baseline CORT also included bleed time (which was always less than 3 min) as a covariate as it can influence CORT levels [33]. In addition, we examined the potential effects of the treatment on telomere loss and chick growth between the first and the second sampling points. In these models, in addition to treatment, we also included sex and hatching rank as fixed factors, and brood size as a covariate. To account for random variation in the starting telomere length among the individuals in each group, telomere length at day 10 was also included as a covariate in the model of telomere loss. Telomere loss was calculated as the daily change in telomere length (telomere length at sampling point 2 – telomere length at sampling point 1/number of days between measurements). Chick growth rate was calculated as the daily mass gain (mass at sampling point 2 – mass at sampling point 1/number of days between measurements). Growth and telomere loss were measured during the period of rapid, linear mass gain in shag chicks. We used repeated measures LME to examine within-individual changes in CORT levels in response to capture and restraint stress and within-individual changes in telomere length. Both CORT levels and telomere lengths were natural log transformed to improve normality. We used a backward elimination process and excluded variables with *p* > 0.05. Least significant difference (LSD) post-hoc tests were used to test for significance between groups. All statistical analyses were performed in IBM SPSS Statistics 19.

## 3. Results

### (a) Corticosterone levels pre- and post-treatment

At the start of the experiment (on average, 10 days after the first chick hatched in each study nest), baseline CORT levels were not significantly different among chicks allocated to the three treatment groups (*F*_2,36.56_ = 2.09, *p* = 0.14, overall mean ± 1 s.e.m.: 27.02 = ± 2.86 ng ml^−1^). Ten minutes after treatment administration, the birds given a CORT dose (handled-CORT birds) had significantly higher CORT levels than those handled and given oil only (handled-oil) (*F*_1,24.99_ = 20.03, *p* < 0.001, mean ± 1 s.e.m.: handled-oil = 56.56 ± 7.56 ng ml^−1^, handled-CORT = 105.53 ± 6.76 ng ml^−1^). Thirty minutes post-dosing or handling, the CORT levels of handled-oil and handled-CORT birds were no longer significantly different (*F*_1,18.00_ = 0.398, *p* = 0.54, mean ± 1 s.e.m.: handled-oil = 55.06 ± 16.38 ng ml^−1^, handled-CORT = 44.40 ± 8.66 ng ml^−1^), though still significantly higher than the baseline mean.

**Figure 1.**
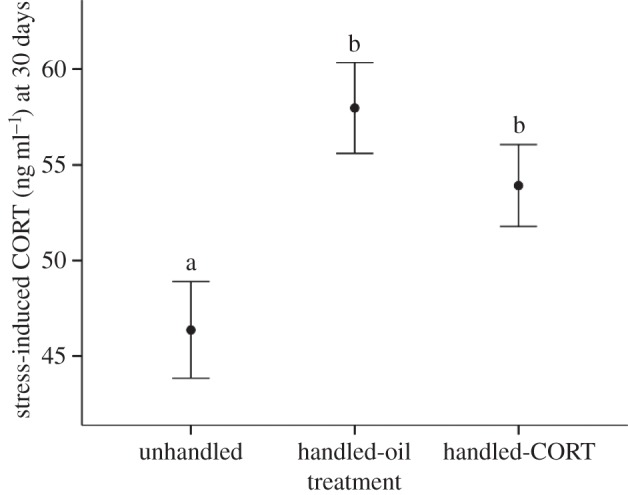
The mean (±s.e.m.) stress-induced CORT levels collected 10 min post-capture and restraint in 30-day-old European shag chicks. All of the chicks within a nest received the same treatment. Unhandled chicks were not visited between days 11 and 29 (*n* = 36 chicks from 17 nests), handled-oil chicks were visited daily and given an oral dose of fish oil only (*n* = 36 chicks from 16 nests) and handled-CORT chicks were visited daily and given an oral dose of CORT suspended in fish oil (*n* = 42 chicks from 18 nests). LSD post-hoc analysis was used to compare differences between groups, and groups that were significantly different at *p* < 0.05 are indicated by different letters.

On the day following the cessation of the treatment, when the first hatched chicks in the nests were around 30 days old, we examined the effects of the treatment on baseline CORT and stress-induced CORT (collected 10 min after capture). There were no differences among the treatment groups in baseline CORT (*F*_2,44.78_ = 0.16, *p* = 0.86; overall mean ± 1 s.e.m.: 15.78 = ± 1.26 ng ml^−1^). As not all chicks in a nest had hatched on the same day, there were slight differences in age among the chicks (range 7 days). Baseline CORT was significantly lower in the chicks that were slightly older at the time of measurement; *F*_1,101.66_ = 6.27, *p* = 0.014), but sex, hatching rank, body mass and bleed time were not significant (all *p* > 0.15). Individual CORT levels increased significantly in response to the 10 min of capture and restraint stress (*F*_1,98.42_ = 570.60, *p* < 0.001) and the magnitude of this increase differed among the treatment groups (*F*_2,45.79_ = 5.11, *p* = 0.010; figure 1). At the end of the treatment period, unhandled chicks had significantly lower stress-induced CORT than handled-oil (*p* = 0.003) and handled-CORT chicks (*p* = 0.027), while the latter two groups were not significantly different from each other (*p* = 0.36). There were no significant effects of age, sex, hatching rank, brood size or body mass on stress-induced CORT levels (all *p* > 0.10).

### (b) Stress exposure and telomere length

**Figure 2.**
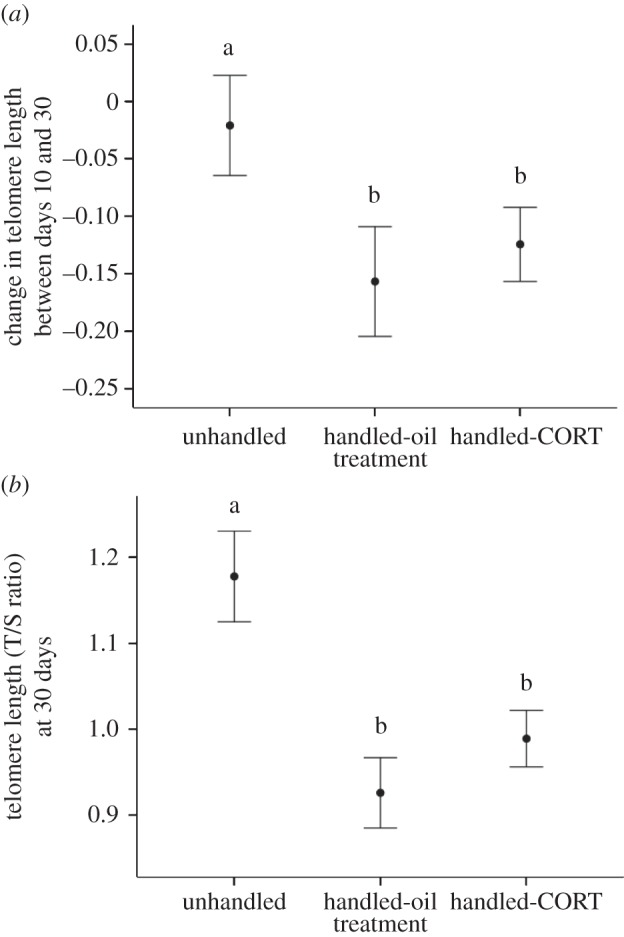
(*a*) The mean (±s.e.m.) change in telomere length calculated as the difference in T/S ratio between days 10 and 30 in European shag chicks. (*b*) The mean (±s.e.m.) telomere length (T/S ratio) in 30-day-old European shag chicks. Telomere length was measured using qPCR and the ratio (T/S) is telomere repeat copy number (T) to control, single gene copy number (S), relative to a reference sample. All of the chicks within a nest received the same treatment. Unhandled chicks were not visited between days 11–29 (*n* = 36 chicks from 17 nests), handled-oil chicks were visited daily and given an oral dose of fish oil only (*n* = 36 chicks from 16 nests) and handled-CORT chicks were visited daily and given an oral dose of CORT suspended in fish oil (*n* = 42 chicks from 18 nests). LSD post-hoc analysis was used to compare differences between groups and groups that were significantly different at *p* < 0.05 are indicated by different letters.

At the start of the experiment, telomere length did not differ among individuals allocated to the three treatment groups (*F*_2,49.82_ = 0.55, *p* = 0.581, q-PCR based telomere length: mean ± 1 s.e.m.: 1.03 ±0.03), though it was shorter in chicks that were slightly older at the time this pre-treatment sample was collected (*F*_1,111.48_ = 6.20, *p* = 0.014). This age-related decline in telomere length continued as the chicks grew older over the 20-day period of the experimental treatment (*F*_1,108.25_ = 20.06, *p* < 0.001), but the magnitude of telomere loss over this period differed among the chicks in the three treatments (*F*_2,42.27_ = 4.02, *p* = 0.025; figure 2*a*). The unhandled chicks experienced significantly less telomere shortening between days 10 and 30 than the handled-oil (*p* = 0.01) and handled-CORT chicks (*p* = 0.03); there was no significant difference in telomere loss between the two handled groups (*p* = 0.58). Consequently, at day 30, there was a significant difference among the three treatment groups in telomere length (*F*_2,43.49_ = 5.77, *p* = 0.006; figure 2*b*). Unhandled chicks had significantly longer telomeres than either handled-oil (*p* = 0.002) or handled-CORT chicks (*p* = 0.014), who again did not differ from each other (*p* = 0.44). Telomere length at days 10 and 30, and telomere loss, were not related to sex, hatching rank, brood size or body mass at the time of sampling or to individual variation in baseline or stress-induced CORT levels. There was also no effect of the slight variation in age at the time of sampling on telomere length at the end of the treatment. There was no difference in growth rate among the chicks in the three treatments (*F*_2,48.10_ = 1.55, *p* = 0.22). However, across all treatments, chicks that had grown faster had shorter telomeres at day 30 (*F*_1,101.58_ = 4.03, *p* = 0.048).

## 4. Discussion

Our results demonstrate that early life telomere loss is accelerated by exposure to stressful circumstances. Understanding the factors responsible for intra-specific variation in lifespan is one of the key areas of biological enquiry. These factors are likely to involve a combination of genetic inheritance and environmental circumstances. There has been great interest in the role of early life circumstances in influencing the pattern and pace of deterioration later in life [5]. Telomere length in vertebrate somatic cells influences the replicative potential of cells, and thereby the pattern of cell loss and rate of accumulation of senescent cells with potentially harmful secretory profiles that can increase inflammation. While other factors, such as the capacity to replenish lost cells, will also be important, a number of studies have shown that telomere length in cell populations is linked to the subsequent lifespan of the host in a wide range of taxa [34–38]; where individuals have been followed throughout their lives, and telomere length measured at different ages, telomere length early in life has been found to be the best predictor of eventual lifespan [15].

Exposure to stressors in early life has been found to have long-lasting phenotypic consequences [2,33]. This may give rise to increased stress responsiveness [28,39,40] and thereby increased individual exposure to the damaging consequences of repeated exposure to high levels of stress hormones. Such increased stress responsiveness was induced in the two handled groups in our study. Recently, environmentally generated stress has been found to be associated with reduced telomere length, an effect deemed ‘too toxic to ignore’ [7]. However, experimental demonstration of the effect of stress exposure in early post-natal life on telomere dynamics has been lacking. Our study, in a natural population of wild birds, shows that the age-related decline in telomere length was faster in groups exposed to stressors during growth and development, giving rise to reduced telomere length. Because we measured telomeres in red blood cells the effect we have found is not a consequence of induced changes in populations of immune cells, but must be a more general effect occurring in progenitor cells of the haematopoietic system. We also found that faster growth was associated with increased telomere loss across all treatment groups. However, since we did not experimentally manipulate growth rate, which was not affected by our treatment, this effect could be due to a number of factors including variation in parental quality. Body mass itself was not related to telomere length or loss, nor did we find any effect of sex, despite the fact that males grow faster than females in the study species.

Increased exposure to CORT is associated with increased oxidative stress, and this seems the likely route whereby the stress treatments increased telomere loss [10]. Given that the treatments increased the stress responsiveness of the birds, this would have also increased the overall exposure to stress hormones in the treated birds. Our experimental treatment included two ways of increasing daily stressor exposure for a 20 day period—one involving handling only, and the other involving both handling and experimental administration of CORT. The effects on CORT levels shortly after administration of the treatment were as expected; both groups showed elevated CORT levels relative to baseline, with the CORT-treated group having the higher levels—on average almost double those found in the handled-oil group. However, this higher level of CORT did not produce a corresponding increase in telomere loss, which was not significantly different between the two stress treatment groups. The direct elevation of CORT that our treatments induced was relatively short lived, but it is important not to evaluate the effects of repeated exposure to stressors simply in terms of the duration of immediate effects on circulating CORT levels; the longer lasting consequences of the treatment on general stress responsiveness is likely to play an important role [41,42]. Both stressor treatments gave rise to a similar increase in stress responsiveness, and this will result in a more protracted increase in stress hormone and oxidative stress exposure when the individual is exposed to stressful situations. Interestingly, the higher CORT levels that the CORT administration gave rise to in the CORT-treated birds did not result in a further increase in stress reactivity above that observed in the handled-only group, suggesting that there may be a ‘ceiling’ to the effect of stressor exposure on stress reactivity. The increases in telomere attrition observed are therefore likely to be due not simply to the elevation in stress hormones during the administration of the treatment itself, but to the effect on the stress phenotype of the birds, which was similar between the two treated groups.

In conclusion, our experimental manipulation of stressor levels during growth and development clearly demonstrated that stressor exposure increases post-natal telomere loss. This effect is not a consequence of triggering changes in immune cell populations, but likely to be a more general effect. Increased telomere attrition as a consequence of conditions experienced in early life could therefore give rise to a ‘time bomb’ of reduced longevity in the absence of any obvious phenotypic consequences at the time of the stress exposure.

## Notes

All of the experimental procedures used here were done under licence from the UK Home Office and from Scottish Natural Heritage.
